# Consensus on research priorities for Essex & Herts Air Ambulance: a Delphi study

**DOI:** 10.1186/s13049-021-00835-z

**Published:** 2021-01-29

**Authors:** Sarah McLachlan, Hilary Bungay

**Affiliations:** 1 Faculty of Health, Education, Medicine and Social Care, Anglia Ruskin University, Bishop Hall Lane, Essex CM1 1SQ Chelmsford, UK; 2 Essex & Herts Air Ambulance, Flight House, Earls Colne Business Park, Essex CO6 2NS Colchester, UK; 3 Faculty of Health, Education, Medicine and Social Care, Anglia Ruskin University, Young Street, CB1 2LZ Cambridge, UK

**Keywords:** Air Ambulances, Delphi Technique, Research

## Abstract

**Background:**

Consensus methods such as the Delphi technique have been used widely for research priority setting in health care. Within pre-hospital emergency medicine, such approaches have helped to establish national and international research priorities. However, in a dynamic field such as pre-hospital critical care, it is necessary to regularly review the continued relevance of findings. Further, considering the variability between pre-hospital critical care providers, it is also important to determine priorities at the local level. Essex & Herts Air Ambulance (EHAAT) sought to develop a five-year research strategy that aligns with their clinical work streams and organisational priorities.

**Methods:**

All staff and Trustees were invited to participate in an online Delphi study with three Rounds. The Delphi was administered via email and Online Surveys software. The first Round invited participants to submit up to five research questions that they felt were of greatest importance to EHAAT  to advance the care provided to patients. In Round 2, participants were asked to rate the importance of questions from Round 1, while Round 3 required participants to rank questions that were prioritised in Round 2 in order of importance.

**Results:**

22 participants submitted a total of 86 research questions in Round 1, which were reduced to 69 questions following deduplication and refinement. 11 participants rated the importance of the questions in Round 2, resulting in 14 questions being taken forward to Round 3. Following the ranking exercise in Round 3, completed by 12 participants, a top five research priorities were identified. The question deemed most important was “How does a pre-hospital doctor-paramedic team affect the outcome of patients with severe head injuries?”.

**Conclusions:**

The top five research priorities identified through the Delphi process will inform EHAAT’s research strategy. Findings suggest that there is still work to be done in addressing research priorities described in previous literature.

## Background

Essex & Herts Air Ambulance (EHAAT) is a Helicopter Emergency Medical Service (HEMS) charity that provides enhanced pre-hospital critical care to critically ill and injured patients in Essex, Hertfordshire and surrounding areas  24 hours a day, seven days a week. The EHAAT medical team consists of a pre-hospital care doctor and critical care paramedic, alongside a pilot and co-pilot when the team responds to incidents by air. During night hours and when the aircraft are offline, the medical team responds by rapid response vehicle. As an enhanced care provider, the organisation has capacity to deliver advanced interventions such as pre-hospital emergency anaesthesia, thoracotomy and transfusion of blood products, which are not provided by the ground ambulance service. HEMS organisations such as EHAAT are therefore often described as bringing the hospital to the roadside.

 EHAAT is a research active organisation, striving to continually improve patient care and outcomes through evidence-based practice. EHAAT sought to develop a five-year research strategy that aligns with their clinical work streams and organisational priorities, using a bottom up approach to survey all staff members in the organisation. A consensus-based approach was therefore deemed necessary to capture the views of the staff and identify research questions of importance to the organisation, and thus inform the research strategy.

Consensus-based approaches have been used to set research priorities across the spectrum of healthcare settings, ranging from mental health nursing[1] and assisted dying[2] to pre-hospital emergency medicine.[3–5] Fevang and colleagues published a consensus report on the top five research priorities in physician-provided pre-hospital critical care, following a modified nominal group exercise with a European expert panel.[3] The prioritised areas for research were appropriate staffing and training and impact on outcomes, advanced airway management, definition of timings for key interventions, pre-hospital ultrasound and dispatch criteria for pre-hospital critical care services. However, it is important to note that Fevang et al.’s consensus report was published almost a decade ago. Bache et al. have recently called for a review of these priorities to determine whether they have been addressed and if they remain relevant.[6] This is particularly important in the dynamic field of pre-hospital critical care. Further, priority setting work in this field to date has tended to be at a national or international level.[3–5] As there exists considerable variability between HEMS organisations, for instance in composition of the medical team and the interventions delivered, it may be considered more appropriate to conduct local priority setting work within individual services.

Consensus approaches include the nominal group technique and the Delphi method, both of which have been used previously for priority setting in pre-hospital emergency medicine.[3, 5] The Delphi method provides a structured and iterative approach to building consensus among a panel of respondents, who are usually experts within the field of interest.[7] The method is often characterised by online data collection, anonymity of respondents and provision of feedback after each stage of data collection. Advantages include its ability to guide group opinion towards decision-making, its cost-effectiveness[8] and avoidance of any individual dominating the group.[9] Participants are also free to respond at a time and location that is convenient.

A Delphi approach was therefore chosen for research priority setting for pre-hospital critical care within EHAAT. The aim of the study was to identify a ‘top five’ research questions, to inform the organisation’s five-year research strategy.

## Methods

### Design

A three-round online Delphi study was employed, drawing on the methods of Wynaden et al.’s study of research priorities within mental health nursing.[1].

### Participants

An inclusive approach was taken to participant recruitment and all staff at EHAAT were invited to participate. This included the Executive Team (*n* = 6), Clinical Team (*n* = 53) and Charity staff (Fundraising and Events, Communications, Finance and Lottery, Support and Retail, *n* = 58). The Charity’s Trustees (*n* = 9) were also invited to take part. A total of 126 people were therefore contacted about the study. Permission to contact staff and Trustees was obtained from the Charity’s Chief Executive Officer and Medical Director. All staff and Trustees received a letter of invitation, information sheet and copy of the study consent form via email from SM, who is the Charity’s Research Fellow. The letter of invitation contained a link to the online survey.

### Procedure

Hasson et al.’s (2000) research guidelines for the Delphi method[7] were consulted in planning the study and the procedure was based on that of Wynaden et al.[1] Guidance for conducting and reporting survey research was also followed.[10] The study received ethical approval from the Allied Health, Nursing & Midwifery & Medicine School Research Ethics Panel at Anglia Ruskin University (reference: AH-SREP-18-055). Data were collected using Online Surveys; a tool designed for academic research, education and public sector organisations, which is fully compliant with UK data protection law. Consent questions were presented to participants the first time that they completed a Round, and they were required to indicate agreement with these questions before beginning the survey. Participants were informed that they were free to withdraw from the study at any point but that their data could not subsequently be withdrawn, due to the anonymous nature of the study. There was no requirement for participants to take part in every Round and staff were free to join the study at any stage. Each Round was open for two weeks and a reminder email was sent after one week. As a result of the anonymous nature of the study, reminder emails were sent to all staff.

Participants remained anonymous throughout the study but were asked to provide data on their professional role, length of time working in the pre-hospital environment (for clinical staff), and length of time working for EHAAT. An anonymised study code was self-generated by participants in order to link responses between the Rounds. This also served to avoid participants being asked to provide demographic information for each Round that they completed. The code comprised the last two letters of the participant’s postal code and the day of their birthday.

The aim of the first round was to elicit a pool of research questions. Participants were asked to list up to five research questions that they believed were most important for the organisation to address in order to advance the care provided to patients. Illustrative examples of research questions were provided for participants less familiar with research. These were based on published research outside the field of pre-hospital critical care in order to avoid introduction of bias. Participants were asked to be specific about the population and problem of interest, any intervention or comparison condition, and outcomes or experiences that would be explored. Participants were advised that they may wish to consider the relevance and importance of the questions to the organisation and the urgency of addressing the questions. After two weeks, Round 1 closed and the research team collated and analysed responses.

In Round 2, the refined research questions from Round 1 were presented to participants within the organisation’s three clinical work streams (neurological emergencies, out of hospital cardiac arrest, and massive haemorrhage and trauma) and a 'miscellaneous' category. Participants were asked to rate the importance of each question using a 5-point Likert scale, anchored by ‘1 – Not important’ and ‘5 – Very important’.[5] The survey requested that participants gave higher ratings to questions that they felt were most important to advance the care that the organisation provides. Optional free-text boxes were provided for participants to comment on the importance ratings assigned. Questions with a mean importance rating of 4 (‘Important’) or above were taken forward to Round 3.

The aim of Round 3 was to establish consensus on prioritisation of the most highly rated questions from Round 2. Research questions with mean importance ratings of 4 or above were presented to participants within the four categories. Participants were asked to rank the questions in order of relative importance, assigning a value of ‘1’ to the most important, ‘2’ to the second most important’ and so on. Participants were asked to assign priority rankings to questions *across* the four categories rather than within each category. Optional free-text boxes were provided for participants to offer comments. The five questions with the lowest mean scores (and therefore highest importance rankings) from Round 3 will be taken forward as the organisation’s research priorities.

### Patient and public involvement

The aim of this study was to establish consensus on research priorities amongst EHAAT’s staff and Trustees. Therefore, patients and members of the public were not consulted at this stage but will be involved in shaping the development of ensuing projects and throughout the research cycle.

### Data analysis and statistics

Data from Round 1 were exported from Online Surveys to Microsoft Excel. Descriptive statistics were calculated for length of time working for the organisation and, for clinicians, length of time working in the pre-hospital setting. Multiple submissions of the same or highly similar research question were collapsed into a single question. To increase methodological rigour, two researchers de-duplicated and refined the research questions independently. Discrepancies were discussed and resolved without need for involvement of a third party. The refined questions were then assessed for alignment with the three clinical work streams of neurological emergencies, out of hospital cardiac arrest, and massive haemorrhage and trauma. Research questions which did not fit within the work streams were assigned to a 'miscellaneous' category.

Data from Rounds 2 and 3 were exported from Online Surveys to Excel and then imported to the SPSS Statistics software package version 24.0 for analysis. Descriptive statistics were calculated for length of time working for the organisation and, in the case of clinicians, length of time working in the pre-hospital setting. Continuous data that were normally distributed are presented as means and standard deviations (SD). Continuous data that were not normally distributed are presented as ranges, medians and interquartile ranges (IQR). Categorical data are presented as counts and percentages. Means and standard deviations were calculated for importance ratings in Round 2 and importance rankings in Round 3. Thematic analysis was used to identify themes in the free text responses.

## Results

### Round 1

Twenty-two participants completed Round 1, including three Charity staff, seven pre-hospital care doctors, five critical care paramedics, one member of the Executive Team, two Trustees and four staff with roles spanning two areas of the organisation. This represented a response rate of 17.5 %. The length of time that these participants had been working with the organisation ranged from 3 weeks to 17 years (median = 3.5 years, IQR = 1.0–5.25 years). For clinical staff, length of experience working in the pre-hospital environment ranged from 1 to 30 years (mean = 12.94 years, standard deviation = 7.52 years). A total of 86 research questions were submitted; following deduplication and refinement, 69 questions were taken forward to Round 2. The majority of questions were assigned to the ‘miscellaneous’ category (*n* = 34), while 5 questions related to neurological emergencies, 11 to out of hospital cardiac arrest, and 19 to massive haemorrhage and trauma. A full list of research questions generated in Round 1 is available from the first author on request. A large proportion of questions were clinical in nature, for instance relating to particular interventions or aspects of care, but there were also suggestions for non-clinical research such as qualitative work on the perceptions and experiences of patients.

### Round 2

Eleven participants completed Round 2, including four pre-hospital care doctors, one critical care paramedic, one Trustee, and two staff with roles spanning two areas of the organisation. This constituted a response rate of 8.7 %. As a result of missing data, it was not possible to identify the roles of the remaining three participants. Nine of these participants indicated that they had also taken part in Round 1. For those who provided data (n = 8), length of time working with the organisation ranged from three weeks to 9 years (mean = 3.82 years, SD = 3.08 years) and clinicians’ experience in the pre-hospital setting ranged from 3 to 30 years (mean = 12.57 years, SD = 8.94 years). Descriptive statistics for importance ratings and free text responses for research questions with a mean importance rating of 4.0 (‘Important’) or greater are presented in Table 1. There was representation of each of the clinical work streams in the prioritised questions, with a heavier weighting toward massive haemorrhage and trauma, consistent with the findings from Round 1. Free text responses indicated that research on the accuracy of injury diagnoses and appropriate dispatch of HEMS resources for out of hospital cardiac arrest would be useful to the organisation.

**Table 1:** Mean and standard deviation importance ratings and free text responses for research questions with a mean importance rating of 4.0 (‘Important’) or greater in Round 2

Category	Question	Mean rating	SD rating	Free text responses
Neurological Emergencies	How does a pre-hospital doctor-paramedic team affect the outcome of patients with severe head injuries?	4.18	1.17	
	How accurate are our diagnoses of head injuries? Are patients triaged appropriately to Neurological centres?	4.45	0.52	We may find patterns of patients we are missing.
	For patients with traumatic brain injury (TBI), does pre-hospital anaesthesia and transfer direct to a neurosurgical facility lead to better outcomes than patients with TBI who are intubated in a trauma unit?	4.27	1.19	
Out of Hospital Cardiac Arrest	What is the threshold for sending a HEMS team to an out of hospital cardiac arrest (OHCA)?	4.09	0.94	I think we need to know and decide what we are targeting.
	What is the optimum profile of induction medication for rapid sequence induction (RSI) in different patients in return of spontaneous circulation (ROSC) after cardiac arrest?	4.00	0.89	
	Does post-ROSC intubation and transfer to a Primary Percutaneous Coronary Intervention (PPCI) suite generate better neurological outcomes and long-term survival than those who arrive in a local Emergency Department with a supraglottic airway?	4.00	1.10	
Massive Haemorrhage & Trauma	How sensitive is the ambulance service’s call taking software at identifying a major trauma patient as per the East of England Trauma Network trauma triage tool?	4.09	1.04	
	How can patients with non-compressible haemorrhage be identified during the emergency call?	4.27	0.65	
	Does intubation of profoundly hypovolaemic (Code Red) patients in the pre-hospital setting improve outcome?	4.00	0.89	
	Does the use of predictive tools and alternative RSI drug regimes reduce the incidence of post RSI hypotension in EHAAT trauma patients?	4.20	0.79	
	In awake trauma patients, do HEMS teams do enough to manage pain and temperature throughout the pre-hospital treatment?	4.36	0.92	
	Do trauma patients attended by EHAAT who are transferred to a major trauma centre have improved quality of life compared to similar patients not transferred to a major trauma centre, i.e. transferred to a trauma unit according to the triage tool?	4.45	0.69	
	How accurate is diagnosis of traumatic injuries by EHAAT medical team? A review of working diagnosis of traumatic injuries versus hospital and post mortem records	4.18	0.75	This would be brilliant.
Miscellaneous	Of the calls pre-alerted into hospital by East of England Ambulance Service NHS Trust (EEAST) alone, is there anything the original call details have in common that could have alerted the Critical Care Desk to dispatch HEMS?	4.18	0.75	

### Round 3

Twelve participants took part in Round 3, including three pre-hospital care doctors, three critical care paramedics and four staff members with roles spanning two areas of the organisation. It was not possible to identify the roles of two participants due to missing data. The response rate for this Round was 9.5 %. Nine participants indicated that they had completed at least one earlier Round of the Delphi process. For those who provided data (n = 10), length of time working with the organisation ranged from 8 months to 17 years (median = 4.5 years, IQR = 1.73–6.0 years). For clinicians who completed Round 3, length of experience working in the pre-hospital setting ranged from 3 to 30 years (mean = 14.3 years, SD = 8.25 years). Mean and standard deviation importance rankings and free text responses for the 14 research questions are presented in Table 2. The top five prioritised research questions from Round 3 are shown in bold. While results of Round 2 suggested greater prioritisation of questions relating to the massive haemorrhage and trauma work stream, three of the top five prioritised questions from Round 3 related to neurological emergencies. The questions focused mainly on the impact of HEMS teams and HEMS-specific interventions on patient outcomes. Three of the five questions referred specifically to the impact of pre-hospital advanced airway management/emergency anaesthesia and two to the effect of direct transfer of patients to specialist centres.

**Table 2:** Mean and standard deviation importance rankings and free text responses for the fourteen research questions in Round 3, ordered by highest to lowest mean ranking

Category	Question	Mean ranking	SD ranking	Free text responses
Neurological Emergencies	**How does a pre-hospital doctor-paramedic team affect the outcome of patients with severe head injuries?**	5.00	3.28	
Out of Hospital Cardiac Arrest	**Does post-ROSC intubation and transfer to a PPCI suite generate better neurological outcomes and long-term survival than those who arrive in a local Emergency Department with a supraglottic airway?**	5.50	3.68	Key question to answer.
Neurological Emergencies	**For patients with TBI, does pre-hospital anaesthesia and transfer direct to a neurosurgical facility lead to better outcomes than patients with TBI who are intubated in a trauma unit?**	5.67	4.66	Well established research and evidence that TBIs do poorly in non-specialist centres.
Neurological Emergencies	**How accurate are our diagnoses of head injuries? Are patients triaged appropriately to Neurological centres?**	6.25	3.47	
MassiveHaemorrhage & Trauma	**Does intubation of profoundly hypovolaemic (Code Red) patients in the pre-hospital setting improve outcome?**	6.25	4.27	Already been done.
Massive Haemorrhage & Trauma	How accurate is diagnosis of traumatic injuries by EHAAT medical team? A review of working diagnosis of traumatic injuries versus hospital and post mortem records	6.50	4.34	This is audit.
Massive Haemorrhage & Trauma	Does the use of predictive tools and alternative RSI drug regimes reduce the incidence of post RSI hypotension in EHAAT trauma patients?	6.67	3.87	
Massive Haemorrhage & Trauma	In awake trauma patients, do HEMS teams do enough to manage pain and temperature throughout the pre-hospital treatment?	6.83	3.56	Owe a duty of care to patients to be able to answer this question.
Out of Hospital Cardiac Arrest	What is the threshold for sending a HEMS team to an OHCA?	7.00	3.72	Key question – where do we provide benefit?
Out of Hospital Cardiac Arrest	What is the optimum profile of induction medication for RSI in different patients in ROSC after cardiac arrest?	7.50	3.00	Optimum dose plus safest dose. Needs to be simple. Not all doctors are the same; not all patients are the same.
Miscellaneous	Of the calls pre-alerted into hospital by East of England Ambulance Service NHS Trust (EEAST)alone, is there anything the original call details have in common that could have alerted the Critical Care Desk to dispatch HEMS?	7.83	4.00	
Massive Haemorrhage & Trauma	Do trauma patients attended by EHAAT who are transferred to a major trauma centre have improved quality of life compared to similar patients not transferred to a major trauma centre, i.e. transferred to a trauma unit according to the triage tool?	7.92	4.54	Too complicated. The research will yield nothing.
Massive Haemorrhage & Trauma	How can patients with non-compressible haemorrhage be identified during the emergency call?	8.83	3.64	
Massive Haemorrhage & Trauma	How sensitive is the ambulance service’s call taking software at identifying a major trauma patient as per the East of England Trauma Network trauma triage tool?	9.75	3.60	Greater sensitivity required for both tasking software and trauma triage tool.

The top five prioritised research questions following Round 3 are presented in bold. Questions 4 and 5 had the same mean ranking

Two free text comments suggested that there is already sufficient evidence regarding impact of direct transfer to a neurosurgical facility on patient outcomes following traumatic brain injury, and effect of pre-hospital intubation on outcomes for profoundly hypovolaemic patients. Nevertheless, these questions remained among the top five priorities established in Round 3. One question within the top five priorities constituted audit rather than research (‘How accurate are our diagnoses of head injuries? Are patients triaged appropriately to Neurological centres?’). However, this question was retained because it was viewed as a priority for the organisation and could underpin the development of future research.

## Discussion

The results of this Delphi study will guide EHAAT’s research strategy over the next five years. There is some representation of each of the organisation’s clinical work streams in the top five prioritised questions, but with a heavier weighting on neurological emergencies. Four of the top five questions relate to the impact of HEMS teams or HEMS-specific interventions on patient outcomes. The most highly prioritised question focuses on impact of a pre-hospital doctor-paramedic team on patient outcomes following traumatic brain injury. These questions are complex, due to the multitude of factors involved in patients’ treatment and rehabilitation. Free text comments suggested that there is already sufficient evidence for two of the five questions prioritised in Round 3. However, with regard to the effect of pre-hospital advanced airway management – a component of both of these questions – recent reviews have argued that there is no conclusive evidence regarding impact on mortality.[6, 11] A systematic literature review will be undertaken to identify existing evidence in these areas and steer the direction of further work.

This study employed a structured and rigorous method to establish consensus on research priorities for EHAAT. Literature to date has focused on national and international research priorities for pre-hospital emergency medicine, while the current findings offer a local perspective. An inclusive approach was taken, giving all staff and Trustees within the organisation an opportunity to participate. However, over the course of the three Rounds the participation of non-clinical staff decreased. This was perhaps to be expected, considering the clinical focus of many of the research questions. Nevertheless, the number of participants is comparable to that of previous consensus work in the field.[3] It must also be acknowledged that the findings do not represent the perspective of patients or members of the public. To mitigate against this, patients and the public will be involved in shaping the development of the ensuing research projects and throughout the research cycle. There also remains the need to further operationalise the research questions, particularly in terms of outcomes. This will be done in collaboration with clinicians and patients to ensure that the research remains closely aligned to the priorities of key stakeholders. Bache et al.[6] have suggested that it may be necessary to consider outcomes other than mortality in order to advance our knowledge of the impact of pre-hospital critical care.

## Conclusions

The impact of conveyance to specialist units and pre-hospital advanced airway management on patient outcomes were perceived as key areas requiring further research. This will inform EHAAT’s five-year research strategy.

## Data Availability

The datasets analysed during the current study are available from the corresponding author on reasonable request.

## Abbreviations

EHAAT: Essex & Herts Air Ambulance
HEMS: Helicopter Emergency Medical Service
OHCA: Out of Hospital Cardiac Arrest
PPCI: Primary Percutaneous Coronary Intervention
ROSC: Return of Spontaneous Circulation
RSI: Rapid Sequence Induction
TBI: Traumatic Brain Injury
